# Full mouth rehabilitation with retrievable metal–ceramic implant‐supported fixed prostheses for a young patient with atrophic jaws: a clinical report

**DOI:** 10.1002/ccr3.1112

**Published:** 2017-08-10

**Authors:** Narges Ameri, Marzieh Alikhasi, Vida Rezayani

**Affiliations:** ^1^ Department of Prosthodontics School of Dentistry Tehran University of Medical Sciences Tehran Iran; ^2^ Dental Research Center Dentistry Research Institute Tehran University of Medical Sciences Tehran Iran; ^3^ Department of Prosthodontics School of Dentistry Shahid Beheshti University of Medical Sciences Tehran Iran

**Keywords:** Atrophic edentulous jaws, dental implants, implant‐supported fixed prostheses

## Abstract

Treatment of atrophic edentulous jaws with implant‐supported fixed prostheses is one of the most complicated challenges in dentistry. This clinical report describes the prosthesis which consists of screw retained frameworks with individual cement retained crowns which combines the advantages of the screw retained restoration with the advantage of cement retained.

## Introduction

Dental implants have become important in rehabilitation of partially or completely edentulous patients 1, 2, 3. Although fixed implant‐supported prostheses have achieved predictable high cumulative survival rates 4. Excessive crown height space (CHS) can be considered a risk factor for some mechanical complications of implant‐supported rehabilitations such as screw loosening, abutment, or porcelain fractures 5, 6, 7, 8, 9, 10, 11.

Throughout the years, clinicians and laboratory technicians overcame this limitation through different strategies 1, 2, 4, 5. One is fabrication of metal‐resin restoration 12. Although still in use, this technique has its shortcomings such as fracture of acrylic teeth or loss of the prosthetic screws 13, 14, 15. Another method is using low weight materials such as ZrO2 which can reduce the gravity‐induced loading stress 16. Another strategy to address the mechanical failures is to design individual full contour crowns to be cemented on a screw retained framework 17. In this concept, individual fractured crown can be removed and repaired without the need to remove the entire structure 18, 19.

Nowadays, dentists may face edentulous patients who have atrophic jaws and excessive CHS as a consequence of early age tooth loss*,* periodontitis‐related tooth loss, long‐term edentulism, and use of removable prostheses 20. Dental anomalies such as Amelogenesis Imperfecta (AI) may sometimes lead to early age tooth loss. AI is a group of hereditary disorders characterized by defective formation or calcification of enamel 21, 22. In patients with severe hypocalcified‐ or hypomature‐type AI with impacted teeth, extraction of all unrestorable teeth and rehabilitation with implant‐supported restorations may be the most cost‐effective choice 23.

This clinical report describes step‐by‐step full mouth rehabilitation of a young edentulous patient, who had missed all his teeth because of AI, with retrievable metal–ceramic implant‐supported fixed prostheses.

## Case Report

**Figure 1:**
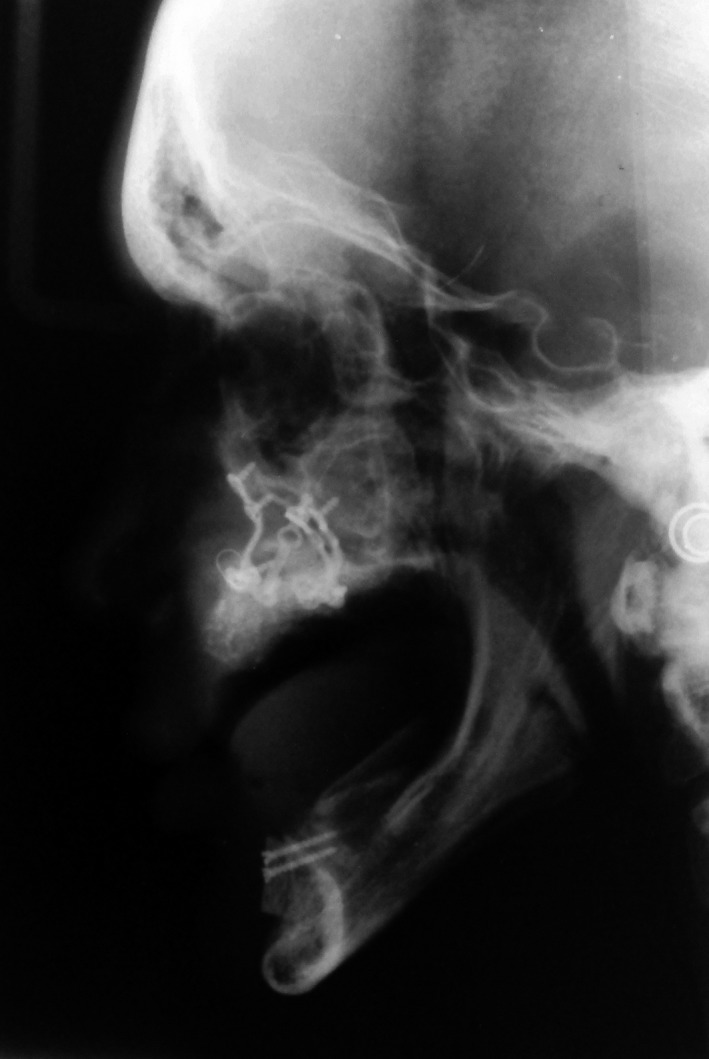
Pretreatment radiographs. Maxillary advancement surgery and augmenting iliac crest bone grafts of both arches.

In 2012, a 22‐year‐old edentulous man applied to the Department of Prosthodontics, Faculty of Dentistry, Tehran University of Medical Science and asked for oral rehabilitation. He had been diagnosed with AI by clinical and radiographic examination and confirmed by the genetic counseling. After a series of restorative treatments, all the teeth had been extracted at age 15, and conventional dentures had been made. The patient had retrognathic maxilla and insufficient bone height and width in both arches, so the maxillary advancement surgery for correction of the jaw relationship and also augmentation of both arches with iliac crest bone graft had been done at age 19 (Fig. 1).

**Figure 2:**
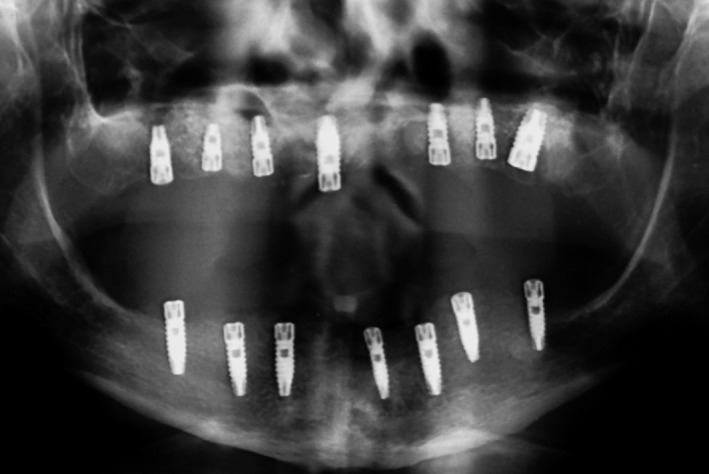
Panoramic radiograph of seven implants in each arch.

After clinical and radiological evaluations, it was decided that the patient was a good candidate to receive implant‐supported prostheses. His existing complete dentures were acceptable considering esthetics, occlusion, and vertical dimension, so they were duplicated as surgical stents. A total of 14 implants (TBR, Connect system, Toulouse, France) with the aid of a laboratory‐ fabricated surgical guide were placed. Seven implants in maxillary arch and seven implants in mandibular arch were placed (Fig. 2). One week later, the dentures were relined with a permanent soft denture liner (Permasoft, Dentsply, York, PA) and inserted in patient` mouth. After 4 months, stage II surgery was done, and soft tissue correction was performed. All implants were approved for loading by the oral surgeon.

After 2 weeks, implant‐level impressions were made with an open tray technique using custom trays, impression copings (TBR, Connect system, Toulouse, France), and polyvinylsiloxane impression material (Panasil, Kettenbach, Huntington Beach, CA). Soft tissue was reproduced with gingival replication material (Soft Tissue Moulage, Kerr, Orange, CA), and master casts were poured with type III dental stone (Microstone, Whip Mix Corp., Louisville, KY). Verification indices were fabricated in pattern resin (GC Pattern Resin LS, GC Corporation, Alisp, IL) on the casts and were checked intraoral.

**Figure 3:**
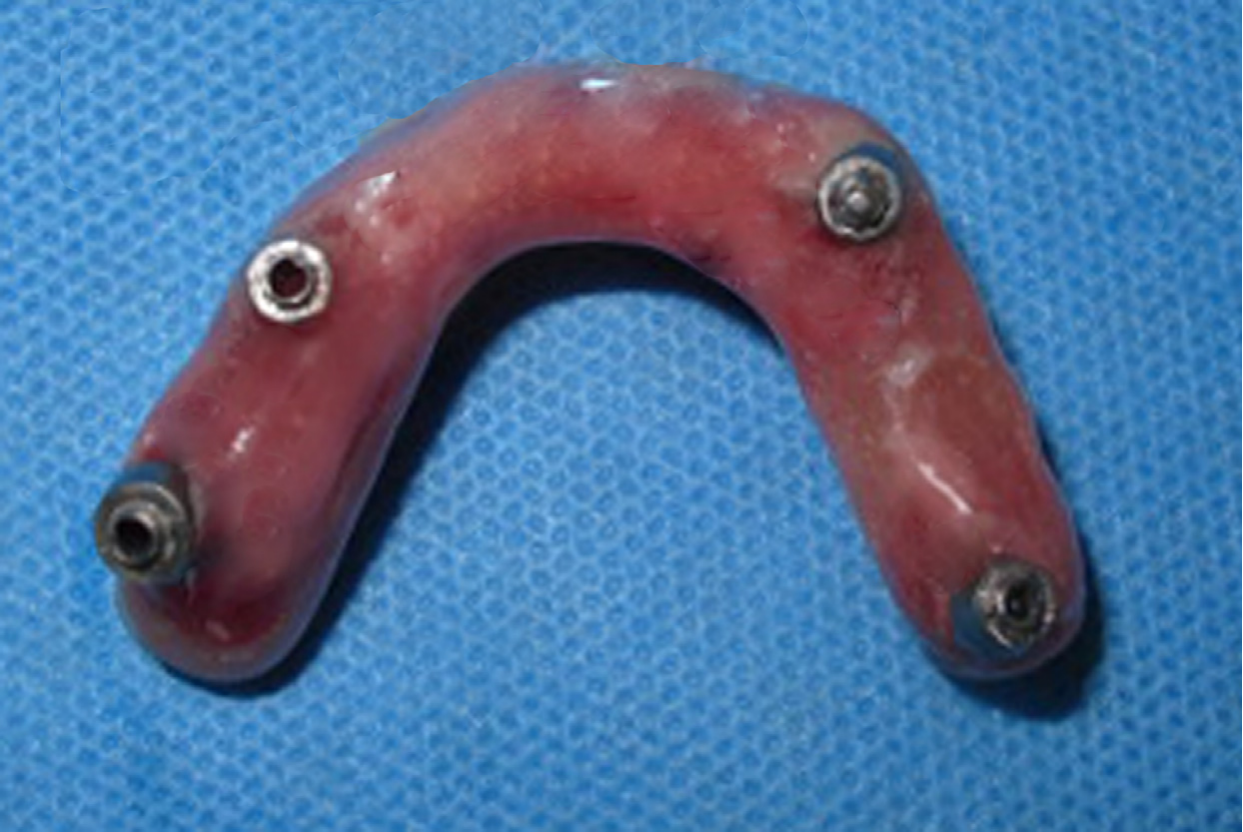
Conversion prostheses.

To evaluate the esthetics without denture flanges, his complete dentures were converted into provisional implant‐supported fixed prostheses (so‐called conversion prostheses). The flanges of prostheses were removed, and the intaglio surfaces were augmented via incremental applications of photopolymerized pink acrylic resin (Astron LC light‐cured hard composite, Astron Dental, Lake Zurich, IL), and then cleaned, and polished (Fig. 3). All coping screws were torqued to 15 Ncm in mouth, and the fit was checked by direct visualization and radiographs. The occlusion was also harmonized for coincidental contacts.

A facebow transfer (HANAU™ Spring Bow, Whip Mix Corporation, Louisville, KY) and an interocclusal record were made using the fixed provisional prostheses. The prostheses were placed on the master casts, and then the casts were mounted on a semi‐adjustable articulator (HANAU™ Wide‐Vue Articulator, Whip Mix Corporation).

Crown height space was measured 17 mm for the maxilla and 18 mm for the mandible according to the silicon matrices of provisional restorations, so the restorations were planned to restore both hard and soft tissues. Considering the path of insertion, a combination of castable screw retained, and screw retained conical abutments were used to produce the optimal angulations.

**Figure 4:**
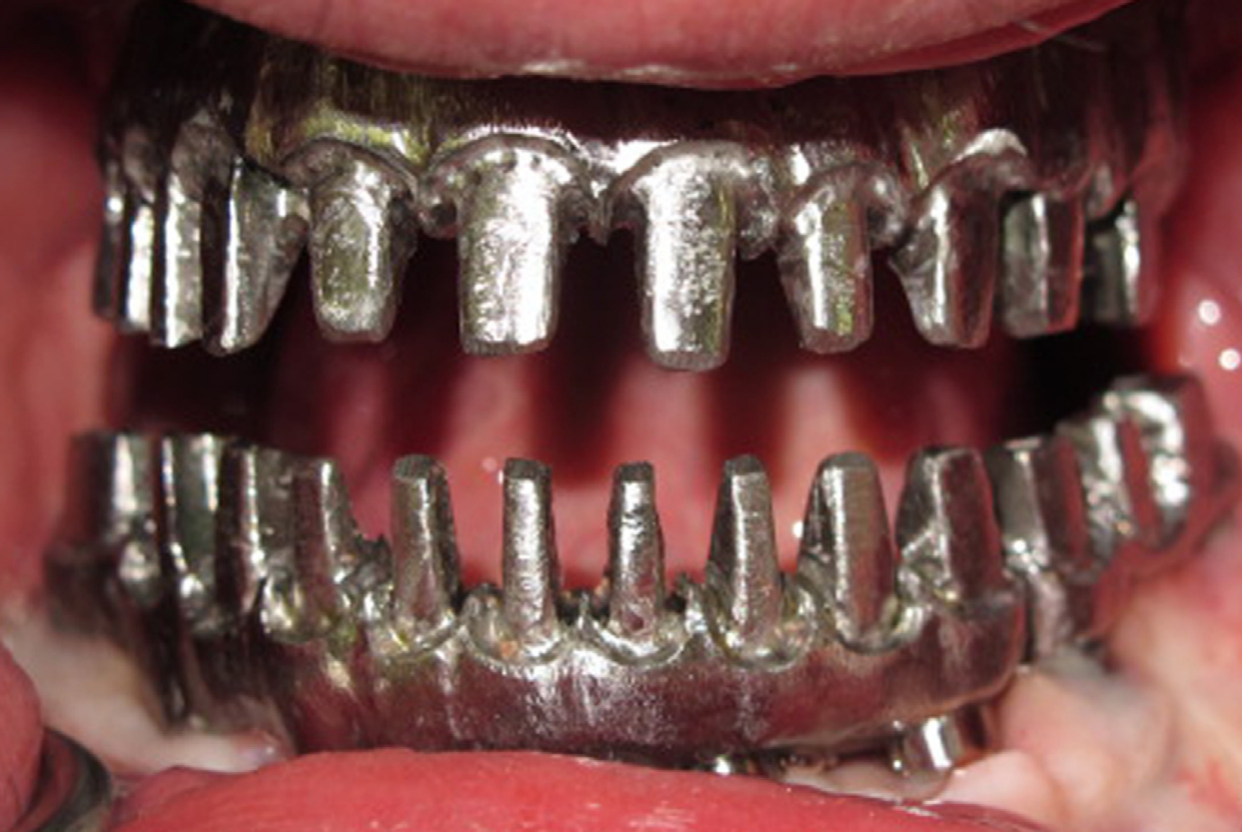
Metal framework.

Resin framework templates (GC Pattern Resin; GC Corporation) were fabricated with individual abutment preparations to accommodate the corresponding individual metal–ceramic crowns. Reduction in the abutment component of the framework allowed for optimal crown thickness, with a minimum of 2 mm. The alveolar portion of each restoration was also cut back so that proper distance could be created for lamination of gingival porcelain material. The mandibular template was sectioned in two pieces to allow mandibular flexion concurrently with jaw movements. The framework templates were casted in a base metal alloy (Palladium‐Silver Alloy; Ivoclar Vivadent, Schaan, Liechtens‐tein). Disclosing media (Kerr's Disclosing Wax; Kerr, Romulus, Mich. and Occlude; Pascal Co Inc., Bellevue, WA.) was used to evaluate the fit of the frameworks on the master casts and to guide adjustment procedures. The fit was refined until the frameworks seated passive on the master casts. The metal frameworks were tried in to evaluate and verify a passive fit intraoral (Fig. 4).

Multiple individual crowns of metal–ceramic were made. The copings were cast in a base metal alloy (Palladium‐Silver Alloy; Ivoclar Vivadent, Schaan, Liechtens‐tein), and the veneering porcelain (Nobel Rondo; Nobel Biocare AB, Kloten, Switzerland) was applied on the copings and fired according to the manufacturer instructions. The metal–ceramic restorations were evaluated to develop a mutually protected occlusion. Gingival porcelain laminations were completed to have slight contacts with mucosa to avoid speech difficulties while permitting access for proper hygiene measures.

**Figure 5:**
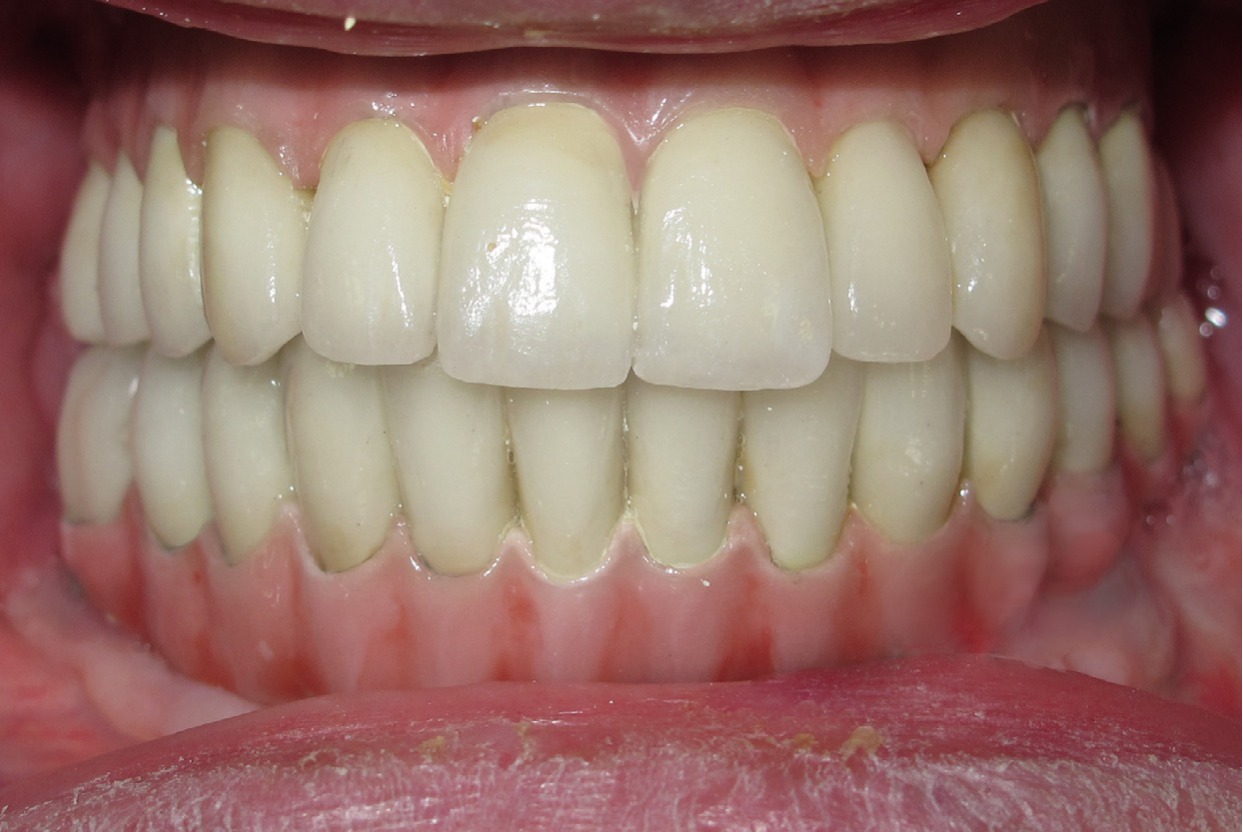
Completed treatment.

**Figure 6:**
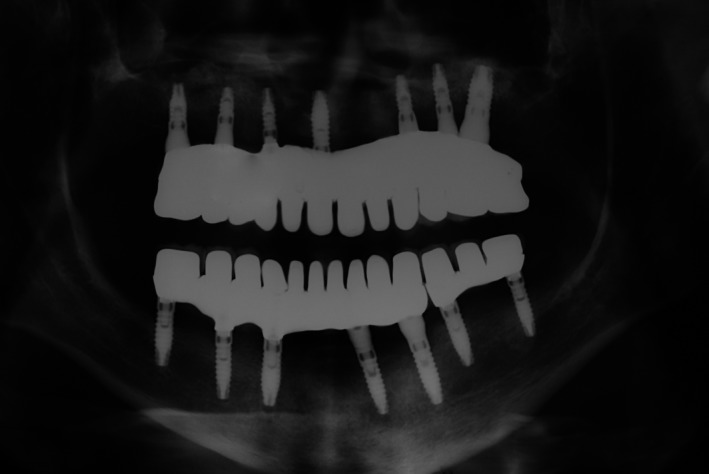
Post‐treatment radiograph.

The prostheses were inserted after staining, glazing, and finishing in dental laboratory. The abutments were screwed in and torqued 20–30 Ncm according to manufacturer recommendation. The screw access holes of the prostheses were sealed with poly tetrafluoroethylene strips, and the crowns were provisionally cemented (TempBond, Kerr, Bioggio, Switzerland). The clinical outcome of treatment and radiographic appearance is shown in Figures 5 and 6. The patient was followed up at regular intervals (every 3 months for first year), and no adverse effect was observed.

## Discussion

It is not preferred for rehabilitation of atrophic jaws with implants to choose metal–ceramic fixed prosthesis. As, this treatment results in a mass of metal that due to its excessive size, weight, and thermal expansion during the application of the porcelain, may be impractical and also, fitness of the casting may be complicated by repeated firing cycles 24. The choice of individual ceramic‐layered crowns cemented on a metal substructure may address to some extent these limitations. On the other hand, this type of prostheses eliminates the screw access openings in the occlusal surface of the crowns and also, makes it possible to remove and repair the fractured porcelain of the individual crown without removing the whole structure 25.

In addition, sealing of the gingival porcelain resembles the esthetics of the anatomical gingival sulcus and allows removal of the excess cement before processing the pink esthetics 26.

## Conflict of Interests

None declared.

## Authorship

NA: involved in study conception and design, treatment of the patient, data acquisition, analysis and interpretation, drafting of manuscript. MA: involved in study conception and design, supervision of treatment, drafting of manuscript, critical revisions. VR: involved in study conception and design, treatment of the patient, data acquisition, drafting of manuscript, critical revisions.
